# Threshold response of stomatal closing ability to leaf abscisic acid concentration during growth

**DOI:** 10.1093/jxb/eru216

**Published:** 2014-05-26

**Authors:** Habtamu Giday, Dimitrios Fanourakis, Katrine H. Kjaer, Inge S. Fomsgaard, Carl-Otto Ottosen

**Affiliations:** ^1^Department of Food Science, Århus University, Kirstinebjergvej 10, DK-5792, Årslev, Denmark; ^2^Institute for Bio- and Geosciences, IBG-2: Plant Sciences, Forschungszentrum Jülich, D-52425 Jülich, Germany; ^3^Department of Agroecology-Crop Health, Århus University, Forsøgsvej 1, 4200 Slagelse, Denmark

**Keywords:** Evaporative demand, grafting, rehydration, relative air humidity, soil water deficit, stomatal closure, stomatal malfunction, stomatal size, transpiration, transpiration efficiency.

## Abstract

Leaf abscisic acid concentration ([ABA]) during growth influences morpho-physiological traits associated with the plant’s ability to cope with stress. A dose–response curve between [ABA] during growth and the leaf’s ability to regulate water loss during desiccation or rehydrate upon re-watering was obtained. *Rosa hybrida* plants were grown at two relative air humidities (RHs, 60% or 90%) under different soil water potentials (–0.01, –0.06, or –0.08MPa) or upon grafting onto the rootstock of a cultivar sustaining [ABA] at elevated RH. Measurements included [ABA], stomatal anatomical features, stomatal responsiveness to desiccation, and the ability of leaves, desiccated to varying degrees, to recover their weight (rehydrate) following re-watering. Transpiration efficiency (plant mass per transpired water) was also determined. Soil water deficit resulted in a lower transpiration rate and higher transpiration efficiency at both RHs. The lowest [ABA] was observed in well-watered plants grown at high RH. [ABA] was increased by soil water deficit or grafting, at both RHs. The growth environment-induced changes in stomatal size were mediated by [ABA]. When [ABA] was increased from the level of (well-watered) high RH-grown plants to the value of (well-watered) plants grown at moderate RH, stomatal responsiveness was proportionally improved. A further increase in [ABA] did not affect stomatal responsiveness to desiccation. [ABA] was positively related to the ability of dehydrated leaves to rehydrate. The data indicate a growth [ABA]-related threshold for stomatal sensitivity to desiccation, which was not apparent either for stomatal size or for recovery (rehydration) upon re-watering.

## Summary

Leaf abscisic acid concentration mediates the growth environment-induced effects on both the control of water loss during desiccation and the restoration of water uptake upon re-watering.

## Introduction

Leaf water status is determined by the balance between water loss and uptake. The loss of water is actively regulated by adjustments in stomatal pore opening (Pantin *et al.*, 2012, 2013). When water loss exceeds water uptake, leaf water potential declines. Low water potentials induce the formation of air bubbles within the xylem vessels (so-called embolism or cavitation), hampering water uptake (Perrone *et al.*, 2012; Brodersen and McElrone, 2013). Functional stomata close in response to dehydration, decreasing water loss and limiting the formation of cavitation (Urli *et al.*, 2013). Upon subsequent water supply, water uptake is restored via embolism refilling (Brodersen and McElrone, 2013). Stomatal responsiveness to desiccation and restoration of water uptake, following a dehydration event, have been related to long-term levels of the hormone abscisic acid (ABA).

Defects in ABA biosynthesis lead to adverse water relations even under conditions of abundant water supply (Finkelstein, 2013). This phenomenon has been linked to impaired stomatal responsiveness to closing cues (Borel *et al.*, 2001; Okamoto *et al.*, 2013). Low leaf ABA concentration ([ABA]), as a result of environmental conditions during growth, also induces attenuated stomatal closing ability (reviewed in Aliniaeifard and van Meeteren, 2013; Fanourakis *et al.*, 2013*b*). A typical example of such a case is low evaporative demand (~0.2 kPa), by means of elevated relative air humidity (RH) (Rezaei Nejad and van Meeteren, 2007; Arve *et al.*, 2013; Giday *et al.*, 2013*a*). The combination of these observations may suggest a causal relationship between long-term [ABA] and stomatal responsiveness to closing stimuli. That is, genetic or environmental factors that promote low [ABA] during growth also reduce stomatal sensitivity to closing signals of fully developed leaves. However, this relationship has so far only been investigated qualitatively by growing plants at two levels of an environmental factor (e.g. RH; Rezaei Nejad and van Meeteren, 2008). Thus a quantitative analysis between [ABA] and stomatal responsiveness (via a dose–response curve) is currently lacking, and the form or strength of this relationship remains unknown.

By using ABA-deficient mutants, it was found that the sensitivity to embolism formation during desiccation is not related to [ABA] (Cochard *et al.*, 1996). However, higher [ABA] exerted a promotive effect on the restoration of water transport via embolism refilling (Secchi *et al.*, 2013). An improved water transport restoration, as a result of higher [ABA], will benefit tissue recovery following water deficit (Brodersen and McElrone, 2013). It has not yet been investigated whether environmentally induced changes in [ABA] affect the recovery of leaf water status during rehydration.

[ABA] is determined by its synthesis and re-distribution within the leaves, but also by the import via the xylem from the roots (Jiang and Hartung, 2008; Daszkowska-Golec and Szarejko, 2013). When reduced [ABA] is due to the aerial environmental conditions (e.g. prolonged periods of low evaporative demand), manipulations in the rhizospheric environment may intensify root-sourced signals and stimulate [ABA]. For instance, deficit irrigation can increase [ABA] (Ackerson, 1980; Davies *et al.*, 2011; Einhorn *et al.*, 2012). Although studies conducted so far have been limited to moderate RH environments (~1 kPa), it may be expected that soil water deficit also elicits an increase in [ABA] at elevated RH.

An alternative method of manipulating root-to-shoot signalling is grafting. However, self-grafts of wild-type plants and wild-type scions grafted on ABA-deficient roots had similar shoot [ABA] (Fambrini *et al*., 1995; Chen *et al.*, 2002). This indicates that ABA synthesis in the root is less relevant in determining [ABA] when ABA can be synthesized in the shoot (Dodd *et al.*, 2009). Interestingly, ABA-deficient scions grafted on wild-type roots showed higher [ABA] compared with ABA deficient self-grafts (Fambrini *et al.*, 1995; Chen *et al.*, 2002). These results suggest that root ABA synthesis can affect [ABA] when shoot ABA synthesis is compromised (Dodd *et al.*, 2009). Genotypes where [ABA] is little affected by high RH have recently been reported (Giday *et al.*, 2013*a*). Therefore, grafting onto roots of these genotypes, showing high [ABA] at elevated RH environments, may stimulate [ABA] under these conditions.

In this study, the quantitative relationship between [ABA] and stomatal responsiveness to leaf water deficit was addressed. Eight long-term [ABA] levels were attained by combining different RH regimes and soil water deficits, as well as by grafting. The effect of [ABA] on the leaf’s ability to recover its weight following a dehydration event was also assessed. It was hypothesized that stomatal functionality is influenced by [ABA] when this is at low concentrations, and that increased [ABA] promotes the recovery of the leaf water potential following water deficit.

## Materials and methods

### Plant material and growth conditions

Experiments included plants of the pot rose cv. ‘Mandarina Kordana’ grown on their own roots, as well as this cultivar grafted onto its own rootstock (self-grafted plants) or onto the rootstock of the pot rose cv. ‘Apache Kordana’. Plants on their own roots (grown from rooted cuttings) were exposed to different levels of both RH and soil water deficit, whereas grafted plants (rootstock of 4-week-old plants; one leaf left on the rootstock stem) were only subjected to different RHs, as explained below. In the remainder of the study, cultivars will be denoted without the (common) second part of their name (e.g. ‘Mandarina’ in place of ‘Mandarina Kordana’). Cultivars were selected based on the contrasting sensitivity of [ABA] to high RH. High RH induced a considerable decrease in [ABA] of ‘Mandarina’, whereas [ABA] was little affected by growth at high RH in ‘Apache’ (Giday *et al.*, 2013*a*).

Following sieving (4mm), 0.55 litre pots were filled by weight (300g per pot) with a mixture of peat and perlite (9:1, v/v; Meegaa substrates BV, Rotterdam, The Netherlands). Soil water content (3g g^–1^) at potting was homogeneous within and between pots. A planting density of four plants per pot was used to facilitate the imposition of soil water deficit during early growth stages at high RH, where otherwise very small amounts of water were consumed daily (<0.3g). Plants on their own roots and grafted plants were placed in two growth chambers, at a density of 25 pots m^–2^. Each chamber accommodated two tables, established as plots. Both chambers had the same air temperature (21.3±1.9 °C). In one chamber, the RH was 60±4% (moderate RH), while in the other chamber an RH of 90±3% (high RH) was obtained, resulting in vapour pressure deficits (VPDs) of 0.99±0.06 kPa and 0.28±0.01 kPa, respectively. In each of the RHs, plants on their own roots were exposed to three watering regimes. Irrigation was conducted manually at the onset of the light period daily by using a nutrient solution (pH of 5.5 and electrical conductivity of 2 mS cm^–1^). Control plants were kept well watered (full irrigation) by maintaining the soil at retention capacity. Soil water deficit treatments were realized by supplying 1/2 (soil water deficit level 1) or 1/4 (soil water deficit level 2) of the amount of water transpired by control plants. Reduced irrigation was applied after plant establishment (from the fourth day onwards). Grafted plants were maintained well watered (full irrigation). Plants on their own roots, experiencing different watering regimes, and grafted plants were randomly distributed in each plot, surrounded by border plants (adjacent to chamber walls) that were not sampled. Irradiance was provided by fluorescence lamps (HQI-BT 400W/D pro; Osram, Munich, Germany) at 400±15 μmol m^–2^ s^–1^ photosynthetic photon flux density (PPFD; determined by LI-250A; LI-COR, Lincoln, NE, USA) for 18h d^–1^. Air temperature, RH [Humitter 50U/50Y(X); Vaisala, Helsinki, Finland], and soil water potential (ES-CVR; TensioTechnik, Geisenheim, Germany) were continuously measured by sensors and data were automatically recorded by loggers. The experiment was repeated once.

Transpiration efficiency (plant level) was determined in growing plants, whereas all remaining measurements (leaf level) were conducted on fully expanded leaves. Leaves were collected at the fifth week following the onset of the experiment, where plants were fully grown (defined as bearing at least two flower buds with cylindrical shape and pointed tip). These leaves were selected from the uppermost sunlit canopy layer. The time between sampling and the start of the evaluation did not exceed 15min.

### Transpiration efficiency throughout growth

The effect of different levels of both RH and soil water deficit on transpiration efficiency [TE; plant mass per transpired water (Thompson *et al.*, 2007)] was evaluated throughout growth. Leaf area and plant (shoot plus root) dry mass were destructively assessed before transferring the cuttings to the growth chambers, as well as weekly following the onset of the treatments for 4 weeks. Mean leaf area and mean plant biomass were calculated for each day, using linear extrapolations between the destructively determined points. By the end of the experiment, plants were fully grown. Leaf area was measured with a leaf area meter (LI-COR Model Li-3100) and dry weight was determined after drying the tissue in a forced-air oven (80 °C) for at least 48h. Pot water loss was determined daily by weighing (±0.1g; MXX-2001; Denver Instruments, Bohemia, NY, USA) the pots, as well as the supplied nutrient and drainage solutions throughout growth. Soil evaporation was assessed daily in four pots without plants but with identical soil water potential to the pots containing plants. Direct evaporation from the soil was subtracted from pot water loss to determine plant water loss. Plant transpiration rate was calculated per unit leaf area. Plant transpiration rate was assessed in 12 plants (four plants per pot); plant mass and leaf area were determined in eight plants after 0, 10, and 17 d or in 12 plants after 24 and 31 d per treatment.

### Stomatal and pore anatomy

The effect of growth conditions on stomatal length (i.e. longest diameter), width (i.e. shortest diameter), and density (i.e. number per unit leaf area), together with pore area (Arve *et al.*, 2013) was determined. The silicon rubber impression technique was employed (Giday *et al.*, 2013*b*) using a lateral leaflet of the first penta-foliate leaf (counting from the apex). Images were acquired using an optical microscope (LeitzAristoplan; Ernst LeitzWetzlar GmbH, Wetzlar, Germany) connected to a digital camera (Nikon DXM-1200; Nikon Corp., Tokyo, Japan). The abaxial (lower) leaflet surface was assessed, since the studied species is hypostomatous (Fanourakis *et al.*, 2013*a*). Sampling took place 2h following the onset of the light period, because this time is required for plants exposed to prolonged darkness to open their stomata and reach a steady-state operating stomatal conductance (Drake *et al.*, 2013). Stomatal (length, width) and pore (area) anatomical features were determined on 25 randomly selected stomata (magnification ×200), and stomatal density was counted on five non-overlapping fields of view per leaflet (magnification ×100). Stomatal size was defined as stomatal length multiplied by stomatal width (Drake *et al.*, 2013). Image processing was performed with the UTHSCSA ImageTool program (University of Texas Health Science Centre, San Antonio, TX, USA). Nine leaflets (one leaflet per plant, and one plant per pot) were assessed in all treatments, with the exception of the self-grafts.

### Stomatal responsiveness to desiccation

The effect of growth conditions on stomatal closing ability in response to desiccation was investigated. Terminal leaflets of the first and second five-leaflet leaves (counting from the apex) were used. Leaflets were detached, re-cut by submerging their petiole under water (to prevent cavitation of xylem vessels that were opened by cutting), and placed in flasks filled with degassed water. The leaflets were incubated at 21 °C, ~100% RH (i.e. VPD close to 0), and under 15 μmol m^–2^ s^–1^ PPFD for 1h to establish their maximum fresh weight (Fanourakis *et al*., 2012, 2013a). The leaflets were then placed on a bench (down*-*facing abaxial surface) in the test room, and the transpiration rate was recorded for 4h by weighing. Test room conditions were air temperature of 21.5±1.7 °C, RH equal to 51±5% (i.e. VPD=1.22±0.03 kPa), and 50 μmol m^–2^ s^–1^ PPFD provided by fluorescent lamps (T5 fluorescent lamp; GE lighting, Cleveland, OH, USA). At the end of the measurement, leaflet area and dry weight were determined, as described earlier. Leaflet relative water content (RWC) was calculated using the following equation (Slavik, 1974):

$$\text{RWC}=\frac{\text{fresh weight}–\text{dry weight}}{\text{saturated fresh weight}–\text{dry weight}}\times \text{1}00$$ (1)

Measurements were carried out on 12 leaflets (one leaflet per plant, and one plant per pot) per treatment. Plants exposed at different RHs and soil water deficits, as well as grafted plants, were assessed.

### Rehydration ability following a dehydration event

The effect of growth environment on the leaf’s ability to regain weight, lost as a result of desiccation, was investigated. Terminal leaflets were collected, and the same procedure as applied for desiccation was followed, as described above. Leaflets were allowed to dehydrate to 85, 70, 55, or 40% of the saturated fresh weight (corresponding to 80±0.5, 63±0.7, 45±1.5, and 30±1.4% RWC, respectively). Then the petioles were immediately placed in flasks filled with degassed water. The leaflets were then incubated for 12h in the rehydration environment (VPD close to 0), as explained above, under darkness. The light was then turned on (15 μmol m^–2^ s^–1^ PPFD) for 2h, while leaflets were still under rehydration conditions. Subsequently, leaf fresh and dry weight was measured.

The rehydration method used was independent of the stomatal component. Leaflet weight was established by the balance between water uptake (through the petiole) and water loss (through transpiration). The latter was minimized by applying a very low VPD and darkening. In this way, growth environment-induced differences in stomatal opening do not result in different rates of water loss during evaluation, which would affect leaflet weight.

Measurements were conducted on 15 leaflets (one leaflet per plant, and one plant per pot) per treatment. Control plants (well-watered) and plants receiving 1/4 of the amount of water transpired by control plants (soil water deficit level 2) at each RH were sampled.

### [ABA] during growth

The effect of growth conditions on [ABA] was determined. First and second five-leaflet leaves (counting from the apex) were collected at 2h following the onset of the light period (Giday *et al.*, 2013*a*), immediately frozen in liquid nitrogen, and stored at –80 °C for further analysis. Liquid nitrogen-frozen samples were freeze-dried, ground, and homogenized (Genogrinder 2000; SPEX SamplePrep, Metuchen, NJ, USA). Deuterated internal standard (2.4ng ml^–1^) was then added to the homogenized samples, before extraction with an accelerated solvent extraction system (ASE 350; Dionex, Hvidovre, Denmark) as described by Pedersen *et al.* (2011). All extractions were duplicated and extracts were diluted with an equal volume of water before analysis.

Chromatographic separation was performed using an HPLC system (Agilent 1200; Agilent, Horsholm, Denmark) equipped with a 150×2.1mm column (Kinetex 2.6 μm PFP, 100Å; Phenomenex, Macclesfield, UK). Gradient elution was performed with 7% acetonitrile in 20mM acetic acid (solvent A) and 78% acetonitrile in 20mM acetic acid (solvent B) at a constant flow rate of 200 μl min^–1^ and injection volume of 20 μl. A gradient profile with the following proportions of solvent B was applied [time (min), %B]: (0, 35), (6, 35), (7, 100), (8, 35), and (18, 35). Prior to analysis, the system was equilibrated (8–18min) with 35% solvent B.

The chromatographic system was interfaced to a liquid chromatography triple quadrupole mass spectrometer (SCIEX 3200; Applied Biosystems, Foster City, CA, USA). The analysis was performed using electrospray ionization in negative mode. Multiple reaction monitoring of unlabelled and labelled ABA analogues was based on the 263>153 and 267>156 mass transitions for *cis*-ABA (Sigma-Aldrich, Brøndby, Denmark) and its deuterated analogue (d_4_ *trans*-ABA; Plant Biotechnology Institute of the National Research council of Canada; Saskatoon, SK, Canada), respectively. The retention times for *cis*-ABA and d_4_ *trans*-ABA were 6.25min and 6.07min, respectively. The calibration curve was prepared from seven ABA standard solutions (0.097–6.250ng ml^–1^) into which equal amounts (1.2ng ml^–1^) of internal standard were added. Subsequently, for each standard solution, the analyte area (divided by the internal standard area) was plotted against the known analyte concentration (divided by the internal standard concentration). Data analysis was performed using the Analyst software v.1.5.1 (Applied Biosystems). The limits of detection (232.8 pmol ABA g^–1^ dry weight plant tissue) and quantification (620.8 pmol ABA g^–1^ dry weight plant tissue) were determined based on a recovery experiment, consisting of four replicates (0.0195ng ABA ml^–1^).

In these measurements, the whole leaf (i.e. the five leaflets were pooled together) was analysed. Twelve replicate leaves (one leaf per plant, and one plant per pot) were assessed in all treatments, with the exception of the self-grafts.

### Statistical analysis

Data were subjected to two-way analysis of variance (ANOVA) using R (version 2.14.2; www.r-project.org). For the analysis of the experiment including water deficit treatments, RH was the main factor and irrigation regime was the split factor. For the grafting experiment, the main factor was also RH, while the genotype of the rootstock was the split factor. For each experiment, data of the two plots were pooled for further analysis, because no significant plot effects were revealed by ANOVA (i.e. minimal microsite influence). The RWC after 4h of desiccation versus [ABA] data was fitted with a four-parameter logistic model (y=c+d−c1+(xEC12)b; Equation 2). Treatment effects were tested at the 5% probability level and the mean separation was done using least significant differences based on Bonferroni adjusted LSD (*P*=0.05).

## Results

### Transpiration efficiency throughout growth

**Fig. 1.**
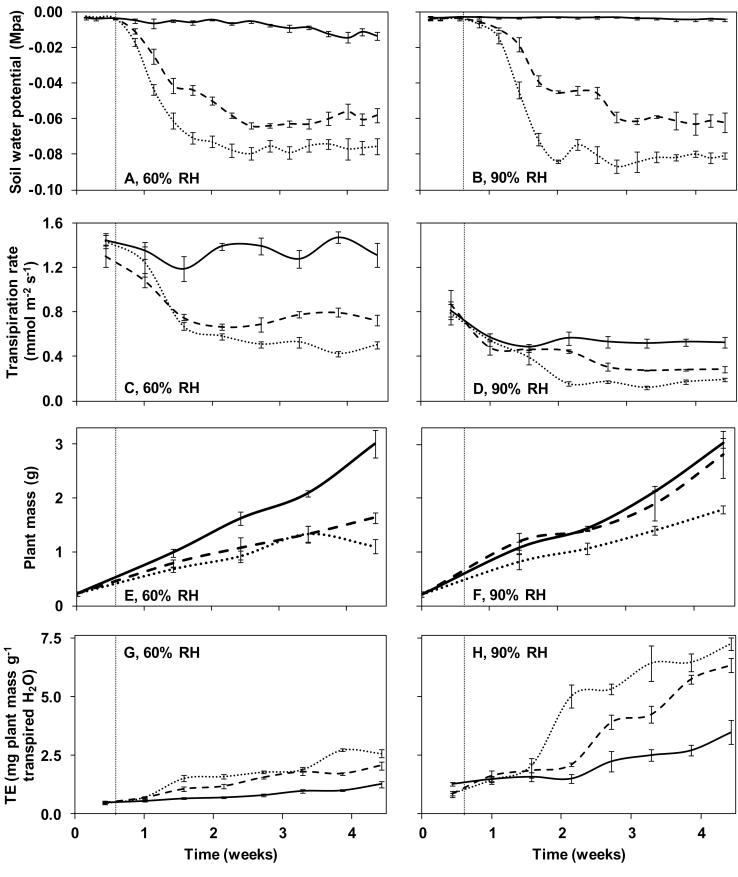
Soil water potential (A, B), transpiration rate (C, D), plant (shoot plus root) mass (E, F), and transpiration efficiency (TE; G, H) during growth at moderate (60%) or high (90%) relative air humidity (RH) of pot rose ‘Mandarina’. Control plants at each RH were maintained well-watered based on evapotranspiration (continuous line). Soil water deficit treatments were realized by adjusting the irrigation to 1/4 (fine dashed line) or 1/2 (dashed line) of evapotranspiration at day 4 (indicated by the vertical line). Soil water potential (*n*=4) and transpiration rate (*n*=12) were assessed daily. Plant mass was determined in eight (0, 10, and 17 d) or 12 (24 and 31 d) plants per treatment. Error bars indicate the SEM.

Three soil water deficit treatments were employed at both RHs. These were realized by adjusting the irrigation amount to 1/4, 1/2, or equal to evapotranspiration throughout growth. In well-watered plants (controls), soil water was readily available (soil water potential ≥ –0.009MPa; Fig. 1A, B). In the soil water deficit treatments, soil water potential decreased progressively, reaching a stable value (–0.060MPa and –0.078MPa, respectively) during the last 2 weeks of growth. The decrease in soil water potential, as a result of deficit irrigation, was accompanied by a lower transpiration rate (per unit leaf area), as compared with controls (Fig. 1C, D). Plants receiving 1/4 of evapotranspiration transpired (per unit leaf area) roughly three times less, as compared with controls, at both RHs.

Besides the evaporative demand, water loss at plant level involves two additional components, a functional (under stomatal control) and a structural (transpiring surface) component (Supplementary Fig. 1A, B available at *JXB* online). Water loss per plant strongly decreased (by up to 78%) due to soil water deficit at moderate RH, with functional and structural components showing similar importance (Supplementary Fig. 1C, E). In contrast to this, the functional component mainly determined the decreased water loss per plant basis of the two soil water deficit treatments at high RH (Supplementary Fig. 1D, F).

Plant (root plus shoot) mass and TE were significantly affected by the interaction between RH and water deficit level (*P*<0.05; Fig. 1E–H). For instance, fully grown plants irrigated with 1/2 of evapotranspiration had 54% lower mass as compared with control plants at moderate RH (Fig. 1E). Instead, the plant biomass was not significantly affected by reducing irrigation to 1/2 of evapotranspiration at high RH (Fig. 1F). The TE ranged over a factor of 6 among the different treatments, showing the highest value in plants receiving 1/4 of evapotranspiration at high growth RH (Fig. 1G, H).

### Stomatal and pore anatomy

For the current evaluation, as well as for the measurements mentioned below, sampling included leaves that were expanded during the last 2 weeks of growth, where soil water potential was nearly stable (Fig. 1A, B).

Stomatal density increased (12%) due to soil water deficit, whereas RH or the rootstock genotype did not produce significant effects (Supplementary Tables S1, S2 at *JXB* online). An interaction between RH and water deficit level was noted for stomatal size. The smallest stomata were observed on leaves of plants receiving 1/4 of evapotranspiration at moderate RH, being 35% smaller than stomata of leaves sampled from well-watered plants at high RH. Stomata of grafted plants (‘Apache’ rootstock) were significantly smaller (11%) than stomata of plants on their own roots. Moderate RH, deficit irrigation, or grafting onto ‘Apache’ rootstock all resulted in smaller (17–51%) stomatal pore areas, with deficit irrigation having the strongest effect.

### Stomatal responsiveness to desiccation

**Fig. 2.**
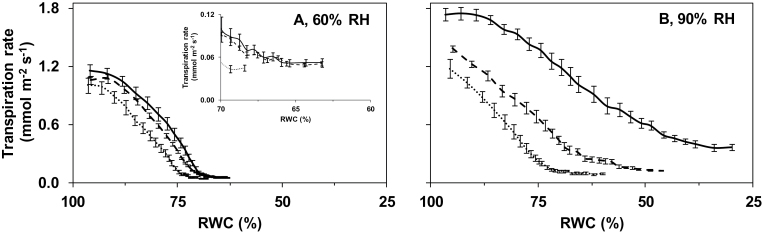
Transpiration rate as a function of relative water content (RWC) during leaflet desiccation of pot rose ‘Mandarina’ grown at moderate (60%, A) or high (90%, B) relative air humidity (RH) under different soil water deficit treatments. Control plants at each RH were maintained well-watered based on evapotranspiration (continuous line). Soil water deficit treatments were realized by adjusting the irrigation to 1/4 (fine dashed line) or 1/2 (dashed line) of evapotranspiration throughout growth (see soil water potential in Fig. 1). Leaflets were left to desiccate for 4h. The insert represents the same data as the main figure, using a different scale. Values are means of 12 leaflets ±SEM.

**Fig. 3.**
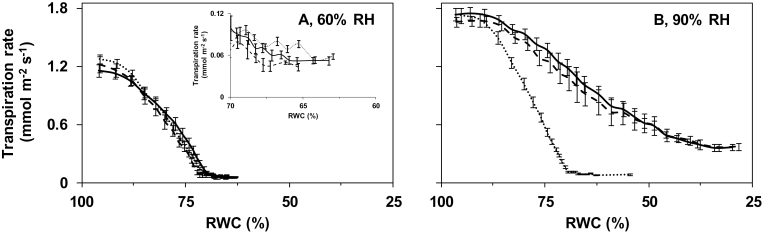
Transpiration rate as a function of relative water content (RWC) during leaflet desiccation of pot rose ‘Mandarina’ grown at moderate (60%, A) or high (90%, B) relative air humidity (RH), and subjected to grafting. Control plants were grown on their own (‘Mandarina’) roots (continuous line). ‘Mandarina’ scions were grafted onto ‘Mandarina’ rootstock (self-grafting; dashed line) or onto the pot rose ‘Apache’ rootstock (fine dashed line). Plants on their own roots and grafted plants were maintained well-watered based on evapotranspiration throughout growth. Leaflets were left to desiccate for 4h. The insert represents the same data as the main figure, using a different scale. Values are means of 12 leaflets ±SEM.

Growth RH-imposed differences in leaf water loss mainly originate from variation in stomatal opening, because the cuticular water loss makes a trivial contribution (Fanourakis *et al.*, 2013*a*). The leaflet transpiration rate in response to desiccation was, therefore, determined to evaluate the effect of growth environment on stomatal opening (Figs 2, 3). In all treatments, the transpiration rate declined as leaflets dehydrated (i.e. with decreasing RWC). Stomata on leaves sampled from well-watered plants cultivated at high RH remained more open than stomata on leaves excised from well-watered plants grown at moderate RH (Figs 2, 3). This led to higher transpiration rates and more dehydrated leaves (i.e. lower RWC) following desiccation.

Soil water deficit during plant growth at moderate RH did not significantly affect stomatal responsiveness to desiccation (Fig. 2A). In contrast to this, deficit irrigation considerably stimulated stomatal responsiveness to desiccation of high RH-grown plants (Fig. 2B). For instance, the RWC at the end of desiccation in plants receiving 1/2 or 1/4 of evapotranspiration was 55% and 100% higher than the respective RWC of well-watered plants at high growth RH. The RWC following desiccation of high RH-grown plants receiving 1/4 of evapotranspiration was not significantly different from the respective value of well-watered plants grown at moderate RH (Fig. 2).

Similarly to soil water deficit, grafting ‘Mandarina’ scion onto ‘Apache’ rootstock did not affect stomatal responsiveness to desiccation at moderate RH (Fig. 3A). However, such grafting enhanced stomatal responsiveness at high growth RH (Fig. 3B). The RWC at 4h after desiccation of leaves sampled from grafted plants was 82% higher than the respective value of plants on their own roots at high growth RH (Fig. 3B). At both RHs, self-grafts (i.e. ‘Mandarina’ scion grafted onto ‘Mandarina’ rootstock) showed the same stomatal responsiveness to desiccation as ‘Mandarina’ plants on their own roots (Fig. 3), indicating that grafting *per se* had no effect.

### Rehydration ability following a dehydration event

**Fig. 4.**
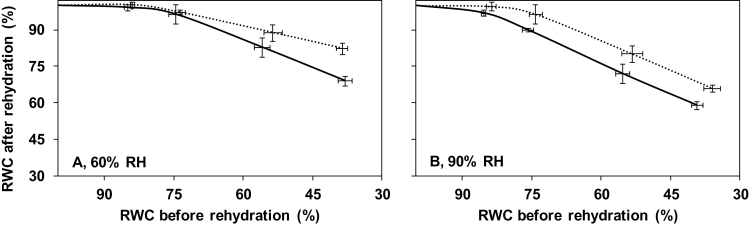
Leaflet relative water content (RWC) following overnight (12h) rehydration, as a function of RWC before rehydration of pot rose ‘Mandarina’ grown at moderate (60%, A) or high (90%, B) relative air humidity (RH) under different soil water deficit treatments. Control plants at each RH were maintained well-watered based on evapotranspiration (continuous line). The soil water deficit treatment was obtained by adjusting the irrigation to 1/4 of evapotranspiration throughout growth (fine dashed line; see soil water potential in Fig. 1). The rehydration method used was independent of the stomatal component. Values are means of 15 leaflets ±SEM.

Leaflets were left to desiccate to a pre-defined RWC (ranging between 30% and 80%), and were subsequently rehydrated overnight. The variation in stomatal opening between treatments (Fig. 2) did not affect the leaf water loss, because evaporative demand during rehydration was minimized. Leaflets desiccated to 80% RWC fully recovered their weight (i.e. dehydration was still reversible) upon rehydration in all treatments, but leaves of well-watered plants cultivated at high RH did not (Fig. 4). Leaflets that were desiccated to RWC values <80% showed partial recovery during rehydration. This recovery was lower in leaves of well-watered plants cultivated at high RH, as compared with leaves of well-watered plants cultivated at moderate RH (Fig. 4). A significant interaction between deficit irrigation and growth RH on fresh weight recovery, following a dehydration event, was noted (*P*<0.001). The promotive effect of deficit irrigation on the recovery of leaf water status was more prominent at moderate as compared with high RH (Fig. 4).

### [ABA] during growth and its relationship with other traits

[ABA] varied over a factor of 5.2 (range 832–4347 pmol g^–1^) between the treatments. Well-watered plants at high growth RH had significantly lower (58%) [ABA] compared with well-watered plants cultivated at moderate RH (Table 1). Both deficit irrigation and grafting (‘Mandarina’ scion onto ‘Apache’ rootstock) triggered an increase in [ABA] at both RHs (Tables 1, 2). This [ABA] increase was statistically significant in all cases, with the exception of the grafting effect at moderate RH. The (relative) increase in [ABA], as a result of either deficit irrigation or grafting, was more prominent at high RH, as compared with moderate RH.

**Table 1.** Endogenous leaflet ABA concentration, expressed on a dry weight (dw) basis, of pot rose ‘Mandarina’ grown at moderate (60%) or high (90%) relative air humidity (RH) under different soil water deficit treatments Control plants at each RH were maintained well-watered based on evapotranspiration. Soil water deficit treatments were realized by adjusting the irrigation to 1/4 or 1/2 of evapotranspiration throughout growth (see soil water potential in Fig. 1). Sampling took place 2h following the onset of the light period.

Irrigation (relative to evapotranspiration)	RH (%)	ABA concentration (pmol g^–1^ dw)
1/4	60	4347 a
	90	1995 c
1/2	60	3627 b
	90	1495 d
1	60	1999 c
	90	832 e
*F* probability		
RH		***
Irrigation		***
RH×irrigation		**

Values are the means of 12 leaves. Means followed by different letters indicate significant differences according to Bonferroni adjusted LSD test (comparison in columns).

**Significant at the 0.01 probability level; ***significant at the 0.001 probability level.

**Fig. 5.**
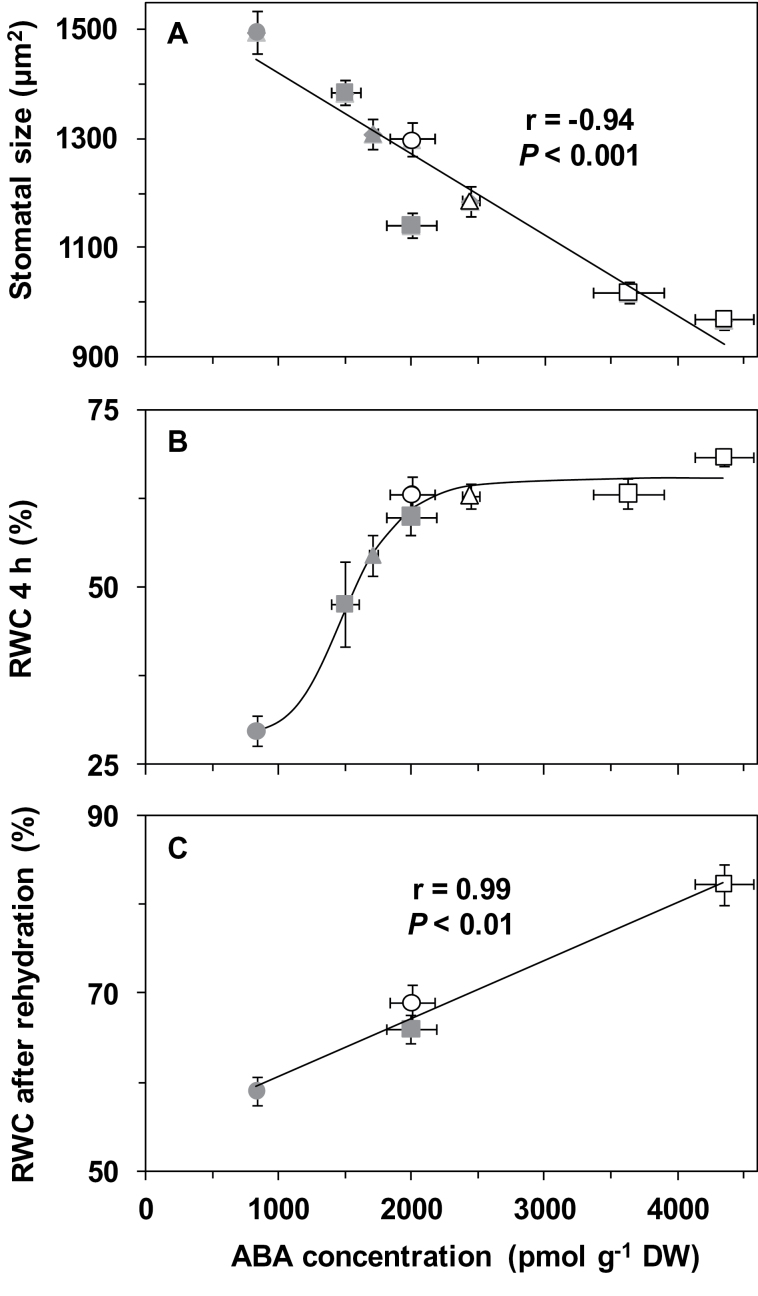
Stomatal size (A; data in Supplementary Tables S1, S2 at *JXB* online), relative water content (RWC) following 4h of leaflet desiccation (B; data of Figs 2, 3), and RWC following overnight rehydration from 30±1.4% (C; data of Fig. 4) as a function of endogenous leaflet ABA concentration, expressed on a dry weight (dw) basis, of pot rose ‘Mandarina’ (data in Tables 1, 12). Plants were grown at moderate (60%, open symbols) or high (90%, filled symbols) relative air humidity, and exposed to different soil water potentials (squares indicate soil water deficit) or subjected to grafting (triangles). The RWC after 4h of desiccation versus [ABA] data was fitted with a four-parameter logistic model (y=c+d−c1+(xEC12)b; Equation 2). All measurements refer to at least 12 replicates, except for stomatal size (*n*=9).

A negative linear relationship between stomatal size and [ABA] was revealed (Fig. 5A). A four-parameter logistic model was fitted to assess the effect of [ABA] on the RWC at the end of desiccation, taken as an indication of stomatal responsiveness. The model-estimated parameters were statistically significant (*P*<0.05). The RWC at the end of desiccation was strongly decreased when [ABA] declined to values lower than a threshold (~2000 pmol g^–1^; Fig. 5B). In contrast to this, the RWC at the end of desiccation was not affected by [ABA], when [ABA] was higher than the (above-mentioned) threshold. In addition, a positive linear relationship between RWC following rehydration and [ABA] was observed (Fig. 5C).

## Discussion

Leaf ABA concentration ([ABA]) is responsive to environmental factors, such as evaporative demand or soil water deficit (Davies *et al*., 2011; Giday *et al.*, 2013*a*). [ABA] during growth, in turn, affects a number of morphological and physiological traits, determining a plant’s ability to endure water deprivation. In this way, growth history can have a considerable impact on plant survival upon exposure to adverse environments (Aliniaeifard and van Meeteren, 2013; Fanourakis *et al.*, 2013*b*). In this study, the effect of long-term [ABA] on the leaf’s ability to control water loss or rehydrate, following water deprivation, was investigated quantitatively.

### Narrower margins of reversible dehydration in plants showing low [ABA]

High RH-grown plants often show disturbed water relations upon transfer to moderate RH environments (Fanourakis *et al.*, 2012; Arve *et al.*, 2013). This wilting phenotype has been related to limited regulation of water loss, as stomata fail to close (Rezaei Nejad and van Meeteren, 2007; Giday *et al.*, 2013*b*). It is shown here that at least one more factor contributes to the adverse water relations of plants grown at high RH. High RH-expanded leaves that were subjected to water deficit had reduced recovery (rehydration) following re-watering, as compared with leaves of plants grown at moderate RH (Fig. 4). This observation indicates that water transport after water limitation is more impaired in plants grown at high RH. The data suggest that the survival of high RH-grown plants following a dehydration event is challenged by both higher rates of water loss (Fig. 2) and a compromised water uptake (Fig. 4).

It was shown here that the recovery (rehydration) following re-watering was closely related to [ABA] (Fig. 5C). In contrast to earlier work, differences in [ABA] were environmentally induced. The embolism formation during desiccation, which impairs water uptake, was found not to be affected by [ABA] (Cochard *et al.*, 1996; Secchi *et al.*, 2013). However, embolism refilling during re-watering, which promotes water uptake, was recently shown to be enhanced by [ABA] (Secchi *et al.*, 2013). Therefore, the observed variation in the recovery following re-watering between leaves with diverse [ABA] (Fig. 5C) is probably related to differences in embolism refilling. Secchi *et al.* (2013) discussed that the promotive effect of [ABA] on embolism refilling seems not to be direct (i.e. on xylem anatomy), but rather related to the stimulation of carbohydrate metabolism. Active metabolism (degradation) of carbohydrates into low-molecular weight osmolytes has been shown to be critical in embolism refilling (Nardini *et al.*, 2011; Brodersen and McElrone, 2013).

Rehydration was here performed under conditions of low evaporative demand to minimize leaf water loss. In this way, differences in stomatal opening between the assessed treatments (Fig. 2) are not expected to affect leaf water balance. Although low evaporative demand during rehydration has been related to a slower rate of embolism recovery, no difference in the degree of embolism recovery between low and high evaporative demands during rehydration was noticeable after 11h (Perrone *et al.*, 2012). Therefore, the low evaporative demand during 12h of rehydration used in this study most probably did not affect the extent of embolism recovery.

### [ABA] stimulates stomatal responsiveness to desiccation, but only up to a threshold

Soil water deficit has been related to increased [ABA] in a wide range of experimental studies, generally conducted under moderate RH conditions (Ackerson, 1980; Dodd, 2009; Davies *et al.*, 2011). It was found here that soil water deficit also triggers an increase in [ABA] at high RH, an environment where [ABA] is very low (Table 1). The low [ABA] at high RH has been related to increased inactivation of ABA rather than decreased synthesis (Okamoto *et al.*, 2009; Arve *et al.*, 2013). The ABA inactivation pathway, however, appears to be species dependent, with conjugation being more important than oxidation in *R. hybrida* plants (Arve *et al.*, 2013). It might be expected that the ABA arriving at the leaf through the transpiration stream is also decreased (due to low transpiration; Fig. 1D), contributing to the low [ABA] of high RH-grown plants. Water deficit has been previously shown to stimulate both within-leaf ABA accumulation (mainly due to decreased catabolism) and ABA delivery to the leaf through the xylem sap (due to increased root ABA synthesis) (Kim *et al.*, 2012; Speirs *et al.*, 2013). At high RH, [ABA] was also increased by grafting (Table 2) onto the rootstock of a genotype previously shown to have high [ABA] under these conditions (Giday *et al.*, 2013*a*). Since self-grafts and plants grown on their own roots had a similar stomatal closing ability (Fig. 3), the observed effects are related solely to the rootstock. Although the transpiration rate during growth was not assessed in plants subjected to grafting, large differences between grafted plants and plants on their own roots are not expected. Therefore, the grafting-induced increase in [ABA] at high RH is most probably due to higher amounts of ABA in the xylem sap due to higher root ABA synthesis.

**Table 2.** Endogenous leaflet ABA concentration, expressed on a dry weight (dw) basis, of pot rose ‘Mandarina’ grown at moderate (60%) or high (90%) relative air humidity (RH), and subjected to grafting Plants were grown on their own (‘Mandarina’) roots or grafted onto the pot rose ‘Apache’ rootstock, and were maintained well-watered based on evapotranspiration throughout growth. Sampling took place 2h following the onset of the light period.

Rootstock	RH (%)	ABA concentration (pmol g^–1^ dw)
‘Mandarina’ (own roots)	60	1999 ab
	90	832 c
‘Apache’	60	2440 a
	90	1780 b
*F* probability		
RH		***
Rootstock		***
RH×rootstock		*

Values are the means of 12 leaves. Means followed by different letters indicate significant differences according to Bonferroni adjusted LSD test (comparison in columns).

*Significant at the 0.05 probability level; ***significant at the 0.001 probability level.

A dose–response curve between stomatal responsiveness and [ABA] was realized here including eight growth scenarios (Fig. 5B). Leaf water status (i.e. RWC) after desiccation was taken as a measure of stomatal responsiveness to desiccation. This was improved in high RH-grown plants, as [ABA] increased up to a threshold (~2000 pmol g^–1^), which is the [ABA] of well-watered plants cultivated at moderate RH. Reduced [ABA] previously has been related to the stomatal malfunctioning of plants cultivated at high RH (Rezaei Nejad and van Meeteren, 2007, 2008; Arve *et al.*, 2013). It was demonstrated here that the relationship between [ABA] and stomatal responsiveness is linear in this [ABA] range (i.e. the linear portion of the sigmoid curve). A further increase in [ABA], however, did not enhance stomatal sensitivity to water deprivation (Fig. 5B). This is in accordance with an earlier report stating that long-term water stress does not affect stomatal sensitivity to the ABA closing stimulus (Peng and Weyers, 1994). Taken together, these results suggest that well-watered plants grown at moderate RH have functional stomata that close in response to desiccation. Growth environment-induced decreases in [ABA], as compared with the above-mentioned condition, result in equal attenuation of stomatal sensitivity to leaf water deficit. In contrast to this, increased [ABA], as compared with well-watered plants exposed to moderate RH, results in a reduced transpiration rate (Fig. 1C), but does not alter stomatal closing ability in response to changes in leaf water status.

### [ABA] mediates growth environment-induced changes in stomatal size

Previous studies have shown that growth conditions affecting [ABA] also influence stomatal size. For instance, high growth RH results in low [ABA] and larger stomata (Rezaei Nejad and van Meeteren, 2007; Arve *et al.*, 2013; Giday *et al.*, 2013*a*). Conversely, drought stress (Xu and Zhou, 2008) or prolonged application of ABA (Fanourakis *et al.*, 2011), which enhance the endogenous ABA content, have been related to smaller stomata. In this study, the effect of [ABA] on stomatal size was investigated in a quantitative manner. It is shown that the relationship between stomatal size and [ABA] is linear and highly significant (Fig. 5A). This relationship suggests that environmental effects on stomatal size are primarily mediated by [ABA].

A few studies have been devoted to relating stomatal size and functioning (see Raven, 2014). It has been suggested that smaller stomata show faster responses between (Drake *et al.*, 2013) and within (Giday *et al.*, 2013*b*) species. It was shown here that water deficit at moderate RH decreases stomatal size, without affecting stomatal responsiveness to desiccation (Fig. 5A, B). These results indicate that stomatal size and responsiveness are poorly related within a genotype.

### Conclusions

The environmental conditions during growth affect both the control of water loss during desiccation and the restoration of water uptake upon re-watering. The current study is focused on the role of [ABA] in mediating these effects. [ABA] varied greatly as a result of plant growth under different levels of soil water deficit at moderate (60%) or high (90%) RH. Grafting onto rootstock of a genotype known to have high [ABA] was also an effective strategy to manipulate [ABA]. The lowest [ABA] was found in well-watered plants cultivated at high RH. Both soil water deficit and grafting triggered an increase in [ABA]. [ABA] was closely related to stomatal size. [ABA] of well-watered plants grown at moderate RH was sufficient to induce functional stomata. Lower [ABA] than this threshold level resulted in a proportional attenuation of stomatal sensitivity to desiccation, whereas higher [ABA] did not produce any permanent effects. However, high [ABA] was still beneficial for leaf water balance, due to its promotive effect on water uptake of previously desiccated leaves.

## Supplementary data

Supplementary data are available at *JXB* online.

Figure S1. Functional (transpiration) and structural (leaf area) regulation of water loss per plant basis during growth at moderate (60%) or high (90%) relative air humidity of pot rose ‘Mandarina’.

Table S1. Stomatal and pore anatomical features of pot rose ‘Mandarina’ grown at moderate (60%) or high (90%) relative air humidity under different soil water deficit treatments.

Table S2. Stomatal and pore anatomical features of pot rose ‘Mandarina’ grown at moderate (60%) or high (90%) relative air humidity and subjected to grafting.

Supplementary Data

## Abbreviations:

ABA: abscisic acid
[ABA]: leaf ABA concentration
PPFD: photosynthetic photon flux density
RH: relative air humidity
RWC: relative water content
TE: transpiration efficiency
VPD: vapour pressure deficit.
