# Supplementary material for: Threshold response of stomatal closing ability to leaf abscisic acid concentration during growth
Source: J Exp Bot. 2014 May 26;65(15):4361–70. doi: 10.1093/jxb/eru216 (PMC4112639; doi:10.1093/jxb/eru216)
Supplement: Supplementary Data [file supp_65_15_4361__index.html]

Threshold response of stomatal closing ability to leaf abscisic acid concentration during growth — Threshold response of stomatal closing ability to leaf abscisic acid concentration during growth — Supplementary Data 

# Threshold response of stomatal closing ability to leaf abscisic acid concentration during growth

## Supplementary Data

Data files

**Files in this Data Supplement:**

- Supplementary Data - Supplementary Data
