# Supplementary material for: Threshold response of stomatal closing ability to leaf abscisic acid concentration during growth
Source: J Exp Bot. 2014 May 26;65(15):4361–70. doi: 10.1093/jxb/eru216 (PMC4112639; doi:10.1093/jxb/eru216)
Supplement: Supplementary Data [file supp_eru216_jexbot116970_file001.pdf]

Supplementary Table 1. Stomatal and pore anatomical features of pot rose 'Mandarina' grown at moderate (60 %) or high (90 %) relative air humidity (RH) under different soil water deficit treatments. Control plants at each RH were maintained well-watered based on evapotranspiration. Soil water deficit treatments were realized by adjusting the irrigation to ¼ or ½ of evapotranspiration throughout growth (see soil water potential in Fig. 1). Measurements took place two hours following the onset of the light period. Per leaflet, five fields of view (stomatal density) and 25 stomata (stomatal and pore dimensions) were analysed. Values are the means of nine leaves. Means followed by different letters indicate significant differences according to Bonferroni adjusted LSD-test (comparison in columns).

Irrigation (relative to evapotranspiration)	RH (%)	Stomatal density (mm^{-2})	Stomatal size (μm^2)	Pore area (μm^2)
	60	63	1096	110[b]
	90	59	1341	193[a]
¼		65[a]	1055	100[b]
½		61[ab]	1202	149[ab]
1		58[b]	1398	203[a]
¼	60	67	969[d]	83
	90	63	1142[c]	117
½	60	65	1019[cd]	88
	90	57	1385[ab]	210
1	60	58	1300[b]	155
	90	58	1496[a]	251
**F probability**				
RH		ns	***	***
Irrigation		*	***	***
RH × Irrigation		ns	*	ns

[ns] Not significant; * Significant at the 0.05 probability level; *** Significant at the 0.001 probability level.

Supplementary Table 2. Stomatal and pore anatomical features of pot rose ‘Mandarina’ grown at moderate (60 %) or high (90 %) relative air humidity (RH), and subjected to grafting. Plants were grown on own (‘Mandarina’) roots or grafted onto the pot rose 'Apache' rootstock, and were maintained well-watered based on evapotranspiration throughout growth. Measurements took place two hours following the onset of the light period. Per leaflet, five fields of view (stomatal density) and 25 stomata (stomatal and pore dimensions) were analysed. Values are the means of nine leaves. Means followed by different letters indicate significant differences according to Bonferroni adjusted LSD-test (comparison in columns).

		Stomatal		Pore
**Rootstock**	**RH (%)**	**density (mm^{-2})**	**size (μm^2)**	**area (μm^2)**
	60	57	1243[b]	143[b]
	90	56	1403[a]	228[a]
‘Mandarina’(own roots)		58	1398[a]	203[a]
‘Apache’		55	1248[b]	168[b]
**F probability**				
RH		ns	***	***
Rootstock		ns	***	*
RH × Rootstock		ns	ns	ns

[ns] Not significant; * Significant at the 0.05 probability level; *** Significant at the 0.001 probability level.

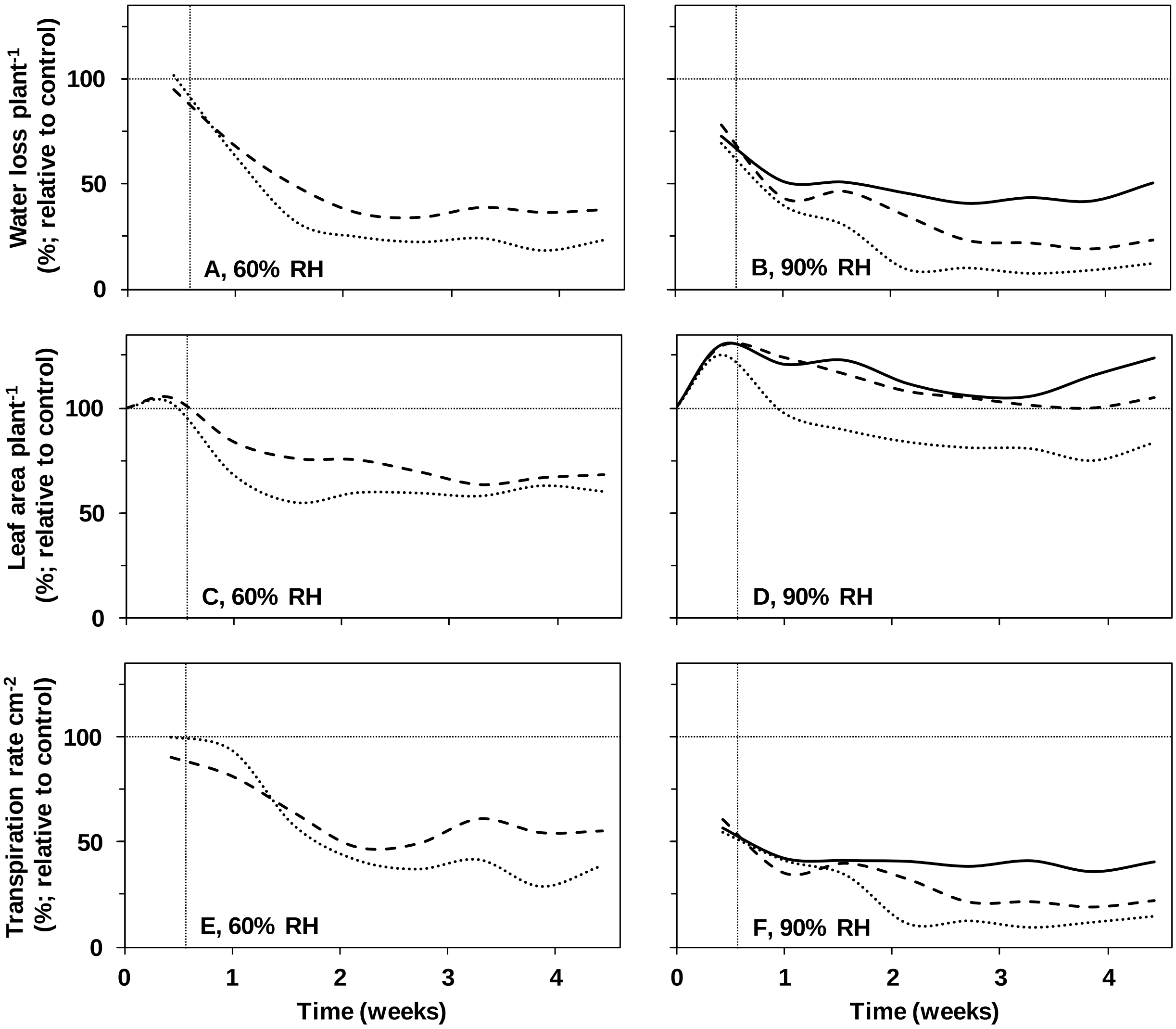

Supplementary Figure S1. Functional (transpiration) and structural (leaf area) regulation of water loss per plant basis during growth at moderate (60%) or high (90%) relative air humidity (RH) of pot rose ‘Mandarina’. Control plants at each RH were maintained well-watered based on evapotranspiration (continuous line). Soil water deficit treatments were realized by adjusting the irrigation to ½ (dashed line) or ¼ (fine dashed line) of evapotranspiration at day 4 (indicated by the vertical line; see soil water potential in Fig. 1). Water loss (A, B), leaf area (C, D) and transpiration rate (E, F) of all treatments was expressed relative to the respective value of control plants (well-watered and 60% RH). Transpiration rate was measured daily in 12 plants, while leaf area was determined in 8 (0, 10 and 17 days) or 12 (24 and 31 days) plants per treatment.
